# G-patch proteins: important regulators of pre-mRNA splicing and ribosome biogenesis

**DOI:** 10.3389/fcell.2026.1750689

**Published:** 2026-04-13

**Authors:** Laura Olivia Karika, Ingrid Cipakova, Lucia Hronska, Lubos Cipak

**Affiliations:** Cancer Research Institute, Biomedical Research Center, Slovak Academy of Sciences, Bratislava, Slovakia

**Keywords:** G-patch protein, *H. sapiens*, pre-mRNA splicing, ribosome biogenesis, RNA helicase, *S. cerevisiae*, *S. pombe*

## Abstract

Pre-mRNA splicing is a fundamental step in eukaryotic gene expression, carried out by the spliceosome. This large and dynamic ribonucleoprotein complex undergoes extensive structural rearrangements during each splicing event. Similarly, ribosome biogenesis is a highly regulated process that requires precise control at every stage, from the transcription of pre-rRNA through its chemical modification and cleavage to the final assembly of mature ribosomal subunits. Central to the regulation of both pre-mRNA splicing and ribosome biogenesis are RNA helicases and their cofactors, notably G-patch proteins. The predominance of G-patch proteins in eukaryotes underscores their evolutionary importance in the increasing complexity of RNA processing and ribosome biogenesis. This review summarizes recent findings on the molecular functions and regulatory roles of various G-patch proteins in the yeasts *S. cerevisiae* and *S. pombe*, as well as in humans. Growing evidence indicates that these proteins act as critical cofactors of RNA helicases involved in splicing, facilitating the dynamic transitions required for spliceosome activation, catalysis, and disassembly. Beyond splicing, these proteins also contribute to the regulation of ribosome biogenesis and other aspects of RNA metabolism. Dysregulation or mutation of G-patch proteins have been shown to cause aberrant mRNA maturation, altered splicing patterns, impaired ribosome assembly, and genomic instability. Such perturbations are associated with a range of human diseases, including cancer progression. Despite the essential roles of G-patch proteins in regulating pre-mRNA splicing and ribosome biogenesis, the precise molecular functions and interaction networks of many G-patch proteins remain poorly understood. Future studies aimed at elucidating the mechanisms by which these proteins coordinate RNA processing and ribosome biogenesis are therefore essential. Such investigations may help uncover the molecular basis of G-patch protein–associated diseases and reveal new potential targets for therapeutic intervention.

## 1 Introduction

G-patch proteins are a family of proteins characterized by a conserved glycine-rich motif known as the G-patch domain. This domain is widespread across eukaryotes but absent in bacteria and archaea, suggesting that it evolved in conjunction with the increasing complexity of eukaryotic gene expression (Aravind and Koonin, 1999). Notably, G-patch domains have also been identified in certain betaretroviruses and human endogenous retroviral elements, although many of these elements are normally transcriptionally silent (Aravind and Koonin, 1999; Garcia-Montojo et al., 2018; Hanke et al., 2016; Jern et al., 2005; Křížová et al., 2012).

In humans, the G-patch protein family includes more than 20 members, many of which contain additional RNA-binding domains, such as RNA recognition motifs, single- or double-stranded RNA-binding domains, zinc fingers, or protein–protein interaction motifs (Fairman-Williams et al., 2010; Robert-Paganin et al., 2015). The diversity in domain composition of these proteins highlights their role in regulating important cellular processes, particularly those that coordinate RNA metabolism (Dardenne et al., 2014; Giraud et al., 2018; Kanwal et al., 2024; Robert-Paganin et al., 2015; Studer et al., 2020). In *S. cerevisiae*, five G-patch proteins have been identified and characterized as regulators of RNA processing, pre-mRNA splicing, and ribosome biogenesis (Chen et al., 2014; Fourmann et al., 2017; Guglielmi and Werner, 2002; Lebaron et al., 2009; Robert-Paganin et al., 2015). In another important model organism, the fission yeast *S. pombe*, nine proteins are annotated as containing a G-patch domain, including orthologs of those found in both *S. cerevisiae* and humans (Lock et al., 2019; McDowall et al., 2015; Rutherford et al., 2024). Recent studies have linked some of these proteins to the regulation of pre-mRNA splicing, suggesting a conserved role for G-patch proteins across eukaryotes (Cipakova et al., 2019; Cipakova et al., 2024; Larson et al., 2016; Portugal-Calisto et al., 2024; Raut et al., 2019; Selicky et al., 2022; Soni et al., 2025).

Notably, recent findings indicate that dysregulation or mutation of G-patch proteins is linked to various diseases. For instance, alterations in G-patch protein expression or their mutations can affect their interactions with RNA helicases, thereby disrupting RNA processing and leading to aberrant gene expression, which in turn promotes tumor progression (Niu et al., 2012; Ren et al., 2025; Studer et al., 2020; Zhang et al., 2022). Dysregulation of G-patch proteins has also been shown to impair mRNA maturation, alternative splicing, ribosome assembly, and genome stability, all of which are essential for cellular homeostasis (Enders et al., 2022; Kanwal et al., 2024; Lebaron et al., 2009; Memet et al., 2017; Niu et al., 2012; Warkocki et al., 2015).

This review summarizes current knowledge on the roles of G-patch domain–containing proteins in the regulation of pre-mRNA splicing across two model organisms, *S. cerevisiae* and *S. pombe*, as well as in humans. Additionally, it highlights recent progress in elucidating the broader molecular functions of these proteins in RNA metabolism, with particular emphasis on their role in regulating ribosome biogenesis.

## 2 Structural features and functional roles of G-patch proteins

**FIGURE 1:**
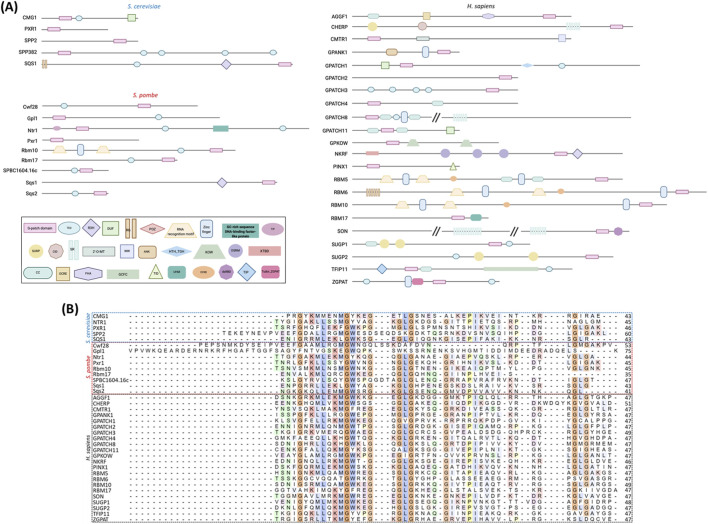
G-patch proteins in *S. cerevisiae*, *S. pombe*, and humans. **(A)** Structure and domain organisation of known G-patch domain–containing proteins in *S. cerevisiae*, *S. pombe*, and *H. sapiens*. **(B)** Alignment of the G-patch domains from *S. cerevisiae*, *S. pombe*, and *H. sapiens* generated using Clustal Omega (Multiple Sequence Alignment) at https://www.ebi.ac.uk/jdispatcher/msa/clustalo. All parameters were kept at their default settings except ORDER, which was set to “input” to preserve the original sequence order in the final alignment.

Despite the G-patch proteins exhibiting considerable diversity in both molecular weight (ranging from 14 to 264 kDa) and domain architecture, they always contain only a single G-patch domain, typically located within an intrinsically disordered region of the protein. Another hallmark of G-patch proteins is the presence of various RNA-binding motifs, such as the KOW motif (Kyprides, Ouzounis, Woese motif), RG/RGG (arginine-glycine/glycine-glycine) and SR (serine-arginine) repeats, and the SURP domain (suppressor-of-white-apricot and PRP21/SPP91), as well as canonical RNA-binding domains including R3H (arginine-3-histidine), dsRBD (double-stranded RNA-binding domain), RRM (RNA recognition motif), and various zinc fingers (Figure 1A). This diverse domain composition reflects the predominant involvement of G-patch proteins in RNA metabolism and highlights their important cellular and regulatory roles in eukaryotes (Heininger et al., 2016).

Notably, G-patch proteins are defined by a presence of a conserved glycine-rich domain, which typically spans 30 to 50 amino acids. This domain was originally identified through analysis of sequence conservation across a wide range of functionally diverse proteins (Aravind and Koonin, 1999). Its consensus sequence Gx_2_hhx_3_Gax_2_GxGlGx_3_pxux_3_sx_10-16_GhG (a – aromatic, h – hydrophobic, l – aliphatic, s – small, u – tiny, x – variable amino acid) features seven highly conserved glycine residues, a strictly conserved aromatic residue following the second glycine, and three distinct hydrophobic patches. Interestingly, while in *H. sapiens* the G-patch domain varies between 46 and 51 amino acids, in *S. cerevisiae* and *S. pombe* the domains span 43–60 and 41–75 amino acids, respectively (Figure 1B). This variation in length suggests potential species-specific adaptations in the structural or functional roles of G-patch proteins.

It has been proposed that the core function of G-patch proteins lies in their ability to regulate the DEAH/RHA-box ATP-dependent RNA helicases (Robert-Paganin et al., 2015; Studer et al., 2020; Tsai et al., 2005; Tsai et al., 2007). This class of helicases is known to use the energy from ATP hydrolysis to unwind RNA structures and remodel ribonucleoprotein (RNP) complexes, enabling dynamic transitions between different spliceosomal states (Bohnsack et al., 2021; Gilman et al., 2017; Jarmoskaite and Russell, 2014). Given the essential role of RNA helicases in pre-mRNA splicing, their activity is tightly regulated (Sloan and Bohnsack, 2018). Importantly, these helicases have been shown to contain autoinhibitory domains that suppress their activity until binding to a specific substrate induces a catalytically active conformation. They also possess domains that recognize distinct RNA features, such as structural motifs or chemical modifications, which help guide the helicase to its appropriate targets (Absmeier et al., 2020; Chakrabarti et al., 2011; Gowravaram et al., 2018; Kretschmer et al., 2018; Luo et al., 2011; Wojtas et al., 2017). However, the predominant mode of their regulation occurs through interactions with cofactor proteins. Among these, G-patch proteins have been found to stimulate their ATPase activity, enhancing their function in RNA processing (Studer et al., 2020; Walbott et al., 2010). Furthermore, structural studies revealed that some G-patch proteins use their G-patch domain to bind to a specific site on RNA helicases, inducing subtle yet critical conformational changes that enhance ATP binding and hydrolysis, thereby priming RNA helicases for unwinding. It has also been suggested that the G-patch domain might function as a flexible molecular brace, linking dynamic regions of RNA helicases and restricting excessive domain movement while maintaining the flexibility required for efficient catalysis (Studer et al., 2020). Together, these findings suggest that G-patch proteins play a crucial role in regulating RNA metabolism, particularly pre-mRNA splicing.

In addition to their role in regulating the pre-mRNA splicing, some G-patch proteins also function as regulators of ribosome biogenesis. In *S. cerevisiae*, G-patch proteins Pxr1 and Sqs1 have been shown to interact with the DEAH-box helicase Prp43. This association stimulates its ATPase activity and facilitates crucial RNA remodeling steps during pre-rRNA processing (Banerjee et al., 2015). It also helps recruit the Prp43 to specific sites within preribosomal particles, thereby ensuring that its activity is spatially restricted. Some G-patch proteins have also been shown to contain additional domains that modulate Prp43 activity through allosteric inhibition (Portugal-Calisto et al., 2024). Similarly, in human cells, some G-patch proteins have been shown to regulate ribosome biogenesis. For example, the telomerase inhibitor PINX1 has been found to bind to helicase DHX15 (the human homolog of *S. cerevisiae* and *S. pombe* helicase Prp43) and to stimulate its ATPase activity, a function conserved from its yeast homolog Pxr1 (Chen et al., 2014). Furthermore, NKRF has been shown to form a ribonucleoprotein complex with helicase DHX15 and the exonuclease XRN2, targeting pre-rRNA spacer regions to promote early rRNA cleavage events (Memet et al., 2017). Recently, another human G-patch protein, GPATCH4, has been shown to stimulate DHX15 helicase activity and bind pre-ribosomal particles, playing a critical role in chemical modification of rRNA and snRNA (Kanwal et al., 2024). These findings clearly support the important role of G-patch proteins in orchestrating the dynamic remodeling events required for ribosome biogenesis.

### 2.1 The role of G-patch proteins in regulating pre-mRNA splicing and ribosome biogenesis

Pre-mRNA splicing is a fundamental step in eukaryotic gene expression. It involves the precise removal of non-coding introns and the ligation of coding exons to produce mature, translatable mRNA. This process is orchestrated by the spliceosome, a multimegadalton macromolecular complex composed of small nuclear ribonucleoproteins (snRNPs) and numerous non-snRNP cofactors (Wahl et al., 2009). During the splicing cycle, the spliceosome undergoes a series of extensive, highly regulated conformational and compositional rearrangements to carry out each splicing event with accuracy and efficiency. These dynamic transitions, including activation, catalysis, and finally disassembly, must be precisely timed and coordinated through the splicing cycle (Fabrizio et al., 2009; Hoskins and Moore, 2012; Senn et al., 2024; Wahl et al., 2009). Among the key factors driving pre-mRNA splicing, RNA helicases and their cofactors, particularly G-patch proteins, have emerged as essential regulatory components (Studer et al., 2020; Walbott et al., 2010).

Interestingly, RNA helicases and their cofactors function not only in splicing but also play crucial roles in other RNA processing pathways, most notably in ribosome biogenesis. Similar to pre-mRNA splicing, ribosome biogenesis is a highly regulated, multistep process. It requires strict control at every stage, from transcription of pre-rRNA, through chemical modification and cleavage, to the final assembly of mature ribosomal subunits (Baßler and Hurt, 2019; Klinge and Woolford, 2019; Kofler et al., 2020; Nazar, 2004). RNA helicases are central to this process, facilitating structural rearrangements, snoRNA release, and the resolution of complex RNA structures within RNPs (Jarmoskaite and Russell, 2014; Kressler et al., 2010; Lang et al., 2024; Sloan et al., 2017; Watkins and Bohnsack, 2012). However, as noted, many RNA helicases exhibit limited substrate specificity and rely on cofactors for precise targeting and regulation. Similar to the role in pre-mRNA splicing, G-patch proteins serve as cofactors in ribosome biogenesis, modulating the activity and specificity of RNA helicases to ensure accurate and efficient ribosomal assembly and biogenesis (Heininger et al., 2016).

#### 2.1.1 G-patch proteins in *S. cerevisiae*

In *S. cerevisiae*, a well-characterized functional interconnection between G-patch domain–containing proteins and RNA helicases involves G-patch proteins and Prp43, a versatile DEAH-box RNA helicase that functions in both splicing and ribosome biogenesis (Table 1) (Bohnsack et al., 2009; Bohnsack et al., 2022).

**TABLE 1:** G-patch domain–containing proteins in *S. cerevisiae*: their sizes, functions, and interacting helicases.

Protein	Size (kDa)	Function	Interacting RNA helicase
Cmg1	32.0	Pre-mRNA splicing	Prp43
Ntr1	83.0	Pre-mRNA splicing	Prp43
Pxr1	31.3	Ribosome biogenesis snoRNPs biogenesis	Prp43
Spp2	20.7	Pre-mRNA splicing	Prp2
Sqs1	86.9	Ribosome biogenesis	Prp43

For instance, the G-patch protein Cmg1 acts as a cofactor of Prp43, stimulating its ATPase activity during splicing (Heininger et al., 2016). In the context of spliceosomal disassembly, another G-patch protein, Ntr1, forms the NTR complex with Ntr2 and Prp43. It has been shown that its G-patch domain alone is sufficient to activate Prp43, while its C-terminal domain functions to prevent inappropriate disassembly of correctly assembled spliceosomes (Christian et al., 2014; Fourmann et al., 2013; Fourmann et al., 2016; Fourmann et al., 2017). Ntr1 has also been shown to mediate the release of the lariat intron from the spliceosome following the completion of the splicing cycle (Tanaka et al., 2007). This step was found to be critical for efficient recycling of spliceosomal components. Additionally, G-patch protein Spp2 is a critical cofactor of Prp2, a helicase that drives the transition from the B^act^ to B* spliceosome. Spp2 has been shown to enhance Prp2’s RNA-dependent ATPase activity and to couple ATP hydrolysis to structural rearrangements within the spliceosome (Bai et al., 2021; Hamann et al., 2020; Krishnan et al., 2013; Roy et al., 1995; Silverman et al., 2004; Warkocki et al., 2009; Warkocki et al., 2015). Together, these findings revealed that in *S. cerevisiae*, the G-patch proteins Cmg1, Ntr1, and Spp2 modulate the localization, activity, and specificity of spliceosomal helicases Prp43 and Prp2, thereby orchestrating key steps in RNA processing.

In contrast to the roles of Cmg1, Ntr1, and Spp2 in regulating pre-mRNA splicing, the last two G-patch proteins in *S. cerevisiae*, Pxr1 and Sqs1, function primarily in ribosome biogenesis. Their G-patch motifs activate Prp43 in a pathway-specific manner (Lebaron et al., 2009). In Pxr1, an inhibitory segment termed the I-patch interacts with Prp43’s catalytic domains to allosterically suppress its ATPase activity. This indicates a dual regulatory mechanism involving both activation and inhibition of Prp43 function (Portugal-Calisto et al., 2024). Furthermore, it has been shown that Sqs1 and Pxr1 associate with 90S pre-ribosomal complexes early in ribosome assembly. Sqs1 has been detected in precursors of both the 40S and 60S subunits, suggesting its involvement in assisting Prp43 throughout the maturation of ribosomal subunits. Sqs1 and Prp43 have also been linked to other factors, including Nob1 and the putative export adaptor Ltv1, which facilitate the transition from 20S pre-rRNA to mature 18S rRNA (Lebaron et al., 2009; Pertschy et al., 2009).

Although the full regulatory network controlling RNA helicases in *S. cerevisiae* has yet to be fully elucidated, it is plausible that their multifunctional roles, ranging from spliceosomal function to ribosome biogenesis, depend on a complex interplay of various cofactors, including G-patch proteins.

#### 2.1.2 G-patch proteins in *S. pombe*

In the fission yeast *S. pombe*, nine proteins are annotated as containing the G-patch domain: Cwf28, Gpl1, Ntr1, Pxr1, Rbm10, Rbm17, SPBC1604.16c, Sqs1, and Sqs2 (Table 2). Four additional, SPAC6F6.19, SPBP4H10.16c, Tma23, and Whi2, are annotated as G-patch–type or G-patch RNA-binding proteins, but without a clearly defined G-patch domain (McDowall et al., 2015; Rutherford et al., 2024; Wood et al., 2012).

**TABLE 2:** G-patch domain–containing proteins in *S. pombe*: their sizes, implicated functions and interacting helicases.

Protein	Size (kDa)	Function	Interacting RNA helicase
Cwf28^a^	44.2	Pre-mRNA splicing	Unknown
Gpl1	60.8	Pre-mRNA splicing Aberrant spliceosomal complex disassembly	Gih35
Ntr1	91.7	Pre-mRNA splicing Spliceosomal complex disassembly	Prp43
Pxr1^a^	31.9	Ribosome biogenesis	Unknown
Rbm10^a^	64.4	Pre-mRNA splicing Heterochromatin assembly	Unknown
Rbm17^a^	33.4	Pre-mRNA splicing	Unknown
SPBC1604.16c	22.6	Uncharacterized	Unknown
Sqs1	79.1	Uncharacterized	Unknown
Sqs2	21.0	Uncharacterized	Unknown

^a^ Function inferred from homology; not yet experimentally validated.

All G-patch proteins in *S. pombe* have human homologs, yet only a few have been studied in detail. Among these, Cwf28 has been suggested to participate in pre-mRNA splicing, as it co-purifies with Cwf14 and numerous associated splicing factors (Kallgren et al., 2014). This role is further supported indirectly by its *S. cerevisiae* homolog, Spp2, which stimulates Prp2’s RNA-dependent ATPase activity and couples ATP hydrolysis to conformational changes within the spliceosome (Bai et al., 2021; Hamann et al., 2020; Krishnan et al., 2013; Roy et al., 1995; Silverman et al., 2004; Warkocki et al., 2009; Warkocki et al., 2015). Another experimentally characterized G-patch protein in *S. pombe* is Gpl1, which forms a functional complex with the putative RNA helicase Gih35 (human DHX35). Gpl1 has been shown to associate with the spliceosome and is required for efficient splicing of a subset of intron-containing transcripts (Aronica et al., 2016; Cipakova et al., 2019; Selicky et al., 2022). Loss of Gpl1 has been shown to cause splicing defects and increased sensitivity to DNA-damaging agents, likely due to impaired processing of pre-mRNAs that encode factors involved in maintaining genome stability (Cipakova et al., 2024). Furthermore, Gpl1 together with Gih35 regulates spliceosome quality control by promoting the disassembly of aberrant spliceosomal intermediates (Soni et al., 2025). Concerning Ntr1, this G-patch protein has been found to play a crucial role in spliceosome disassembly. Similar to its *S. cerevisiae* homolog, which acts as a cofactor for the RNA helicase Prp43 by stimulating its ATPase and helicase activities (Fourmann et al., 2013; Fourmann et al., 2016; Fourmann et al., 2017; Tsai et al., 2005), Ntr1 in *S. pombe* interacts with Prp43 and Ntr2 (Cipakova et al., 2019; Raut et al., 2019). The evolutionary conservation of Ntr1 underscores its fundamental role in RNA metabolism and spliceosome dynamics.

In contrast, several G-patch proteins in *S. pombe* remain poorly characterized. Pxr1´s role in ribosome biogenesis is primarily inferred from its *S. cerevisiae* homolog (Guglielmi and Werner, 2002). Similarly, Rbm10 and Rbm17 are not well studied, though they are suggested to participate in pre-mRNA splicing (Rutherford et al., 2024; Wood et al., 2012). A recent study has shown that overexpression of Rbm10 leads to severe growth defects and widespread intron retention. Interestingly, Rbm10 also appears to contribute to heterochromatin assembly, suggesting a previously unrecognized mechanism by which this G-patch protein may contribute to heterochromatin-mediated gene silencing (Weigt et al., 2021). Finally, the remaining G-patch domain–containing proteins in *S. pombe*, SPBC1604.16c, Sqs1, and Sqs2, have no direct experimental or functional data regarding their molecular functions. Based on evolutionary conservation, however, these proteins are likely to function similarly to their *S. cerevisiae* or human homologs in the regulation of pre-mRNA splicing, ribosome biogenesis, transcription, or cell cycle progression (Huen et al., 2010; Lebaron et al., 2009; Pertschy et al., 2009; Phillips et al., 2024; Sharma et al., 2010; Sharma et al., 2011; Lv et al., 2025).

Despite their evolutionary conservation and the presence of human orthologs, the molecular functions of many G-patch proteins in *S. pombe* remain poorly understood. Therefore, a detailed investigation of their interactomes and functional roles is essential. Given the genetic tractability and conserved RNA processing machinery of *S. pombe*, these investigations not only deepen our understanding of RNA metabolism but also provide valuable insights into the roles of their human counterparts, whose dysfunction is linked to various diseases and pathologies.

#### 2.1.3 G-patch proteins in *H. sapiens*

In humans, most G-patch proteins have been functionally associated with pre-mRNA splicing, primarily through their interactions with spliceosomal RNA helicases (Table 3).

**TABLE 3:** Human G-patch domain–containing proteins: their sizes, functions, interacting helicases and yeast homologues.

Protein	Size (kDa)	Function	Interacting RNA helicase	Homologues in *S. cerevisiae*	Homologues in *S. pombe*
AGGF1	81.0	Pre-mRNA splicing Transcription regulation Angiogenesis	Unknown	Sqs1	Sqs1 Sqs2
CHERP	103.7	Pre-mRNA splicing Alternative splicing	Unknown	-	-
CMTR1	95.3	Pre-mRNA capping	DHX15	-	-
GPANK1	39.3	Uncharacterized	Unknown	-	SPBC1604.16c
GPATCH1	103.3	Pre-mRNA splicing Aberrant spliceosomal complex disassembly	DHX35	-	Gpl1 SPBP4H10.16c Whi2
GPATCH2	58.9	Pre-mRNA splicing	DHX15	-	-
GPATCH3	59.3	Pre-mRNA splicing Alternative splicing Immune response	DHX15	-	-
GPATCH4	50.4	Ribosome biogenesis Regulation of rRNA transcription	DHX15 DDX21	-	Tma23
GPATCH8	164.2	Pre-mRNA splicing Alternative splicing	DHX15	-	-
GPATCH11	33.3	Pre-mRNA splicing Transcription regulation	Unknown	Cmg1	SPAC6F6.19
GPKOW	52.2	Pre-mRNA splicing	DHX16	Spp2	Cwf28
NKRF	77.7	Ribosome biogenesis Transcription regulation	DHX15	-	-
PINX1	37.0	Ribosome biogenesis Telomerase inhibition	DHX15	Pxr1	Pxr1
RBM5	92.2	Pre-mRNA splicing Alternative splicing	DHX15	-	Rbm10
RBM6	128.6	Pre-mRNA splicing Alternative splicing	Unknown	-	Rbm10
RBM10	14.2	Pre-mRNA splicing Alternative splicing	DHX15	-	Rbm10
RBM17	45.0	Pre-mRNA splicing Alternative splicing	DHX15	-	Rbm17
SON	263.8	Pre-mRNA splicing Alternative splicing Transcription regulation	Unknown	Sqs1	Sqs1 Sqs2
SUGP1	72.5	Pre-mRNA splicing	DHX15	-	-
SUGP2	120.2	Pre-mRNA splicing Alternative splicing	Unknown	-	-
TFIP11	96.8	Pre-mRNA splicing DNA repair	DHX15	Ntr1	Ntr1
ZGPAT	57.4	Pre-mRNA splicing Transcription regulation	DHX15	-	-

Among these, RBM5, RBM6, RBM10, and RBM17 have been reported to modulate alternative exon usage and splice-site selection. They have been found to regulate spliceosome assembly and to control the inclusion or exclusion of cassette exons, particularly in genes involved in apoptosis, cell-cycle regulation, and signal transduction (Coomer et al., 2019; Fushimi et al., 2008; Inoue, 2021; Wang et al., 2013; Zhang et al., 2024). RBM5 and RBM6, which are closely related and frequently co-expressed, have been shown to promote pro-apoptotic splicing patterns by facilitating the inclusion of alternative exons in *FAS*, *NUMB*, *Bcl-X*, and *CASP2* (Bechara et al., 2013; Bonnal et al., 2008; Fushimi et al., 2008; Rintala-Maki and Sutherland, 2004; Serrano et al., 2018). The G-patch domain of RBM5 has been demonstrated to directly bind the spliceosomal RNA helicase DHX15 and enhance its helicase activity (Niu et al., 2012). Similarly, RBM10 has been found to associate with DHX15 and to act as a negative regulator of exon inclusion, fine-tuning mRNA isoform diversity and exhibiting tumor-suppressive properties through modulation of genes such as *NUMB*, *TNRC6A*, *Vinculin*, and *CD44* (Bechara et al., 2013; Cao et al., 2021; Hegele et al., 2012; Imagawa et al., 2023; Krishnamoorthy et al., 2025; Serrano et al., 2018). More recently, RBM5 and RBM10 have been identified as components of the U2 snRNPs, where they regulate splicing after branch-site base pairing (Damianov et al., 2024). Furthermore, RBM17 has been characterized as a spliceosomal component involved in alternative splicing (Al-Ayoubi et al., 2012; Corsini et al., 2007; Liu et al., 2013; Sampath et al., 2003). It has been shown to regulate a subset of short introns by functionally substituting for U2AF and to cooperate with SAP30BP during active spliceosome assembly (Fukumura et al., 2021; Fukumura et al., 2023). Collectively, RBM proteins form a dynamic regulatory network that coordinates alternative splicing decisions essential for maintaining cellular homeostasis and development.

Another human G-patch protein involved in the regulation of pre-mRNA splicing is SON. This protein has been shown to localize to nuclear speckles, subnuclear structures enriched in splicing factors (Mattioni et al., 1992; Saitoh et al., 2004). Unlike its yeast homolog Sqs1, which functions in ribosome biogenesis, SON has been found to facilitate splicing by recruiting SR proteins to RNA polymerase II complexes and has been demonstrated to be essential for proper cell-cycle progression and genome stability (Ahn et al., 2011; Battini et al., 2015; Kim et al., 2021; Phillips et al., 2024; Sharma et al., 2010; Sharma et al., 2011).

Other human G-patch proteins, including SUGP1, SUGP2, and TFIP11, have been reported to contribute to spliceosome dynamics as well. SUGP1 has been suggested to play an important role in branch-point recognition and splice-site pairing through interactions with DHX15, SF3B1, and SF1/U2AF, facilitating U2 snRNP recruitment to branch-point regions (Feng et al., 2023; Xing et al., 2025; Zhang et al., 2022; Zhang et al., 2023). SUGP2 has been shown to interact with the splicing regulator PTBP1 and to regulate alternative splicing during mouse spermatogenesis (Moreno-Aguilera et al., 2024; Zhan et al., 2021). Finally, TFIP11, the human homolog of yeast Ntr1, is a stable component of the spliceosomal IL complex (Wen et al., 2005; Yoshimoto et al., 2009). It has been shown to recruit the RNA helicase DHX15 to promote spliceosome disassembly, facilitating intron release and spliceosome recycling (Tannukit et al., 2009; Vorländer et al., 2024; Wen et al., 2008). Recently, TFIP11 has also been found to function independently of DHX15, contributing to U6 snRNP modification and U4/U6·U5 tri-snRNP assembly (Duchemin et al., 2021). Moreover, it has been demonstrated to form a complex with the BLM helicase to regulate RAD51-mediated repair of stalled replication forks, thereby preserving genome integrity (Chen et al., 2024).

Regarding the human G-patch protein ZGPAT, it has been found to contribute indirectly to RNA processing. It has been shown to associate with the 35S assembly intermediate of the U4/U6·U5 tri-snRNP complex within Cajal bodies and has also been demonstrated to interact with DHX15, stimulating its ATPase and unwinding activities (Chen et al., 2017). Because DHX15 is also present in the 35S tri-snRNP, ZGPAT likely activates DHX15 during tri-snRNP assembly, influencing splicing efficiency and fidelity. In addition, ZGPAT has been reported to function as a transcriptional repressor by binding specific DNA sequences and recruiting chromatin-modifying complexes such as NuRD to gene promoters, thereby suppressing transcription (Li et al., 2009; Yu et al., 2010).

A subset of human G-patch proteins, GPATCH1, GPATCH2, GPATCH3, GPATCH4, GPATCH8, and GPATCH11, has been implicated in splicing-related processes and ribosome biogenesis. GPATCH1, similar to its yeast homolog Gpl1, has been shown to interact with the RNA helicase DHX35 (Dybkov et al., 2023; Soni et al., 2025). Deletion of *GPATCH1* or *DHX35* in *C. neoformans* and of *gpl1* or *gih35* in *S. pombe* has been found to increase alternative splicing frequency (Cipakova et al., 2024; Sales-Lee et al., 2021; Selicky et al., 2022). Recently, the GPATCH1–DHX35 complex has been identified as a key factor in the coordinated disassembly of rejected spliceosomes, linking 5′splice-site proofreading with spliceosome turnover (Li et al., 2025). Regarding GPATCH2, this protein has been shown to enhance the ATPase activity of DHX15, indicating its role in RNA unwinding during splicing (Lin et al., 2009). GPATCH3 has been demonstrated to regulate DHX15 activity and splicing fidelity. It has been found to control alternative splicing of immune-related genes such as *CXCR3*, *CD44*, and *FOXP3*, thereby remodeling the tumor microenvironment (Ren et al., 2025). In contrast, GPATCH4 has been implicated in ribosome biogenesis and RNA modification. It has been shown to localize to both the nucleolus and Cajal bodies, where it modulates the ribosomal activity of DHX15 and regulates 2′-O-methylation of rRNA guided by snoRNAs, processes essential for ribosome function and pre-mRNA splicing (Hirawake-Mogi et al., 2021; Kanwal et al., 2024; Kodera et al., 2024). Recently, GPATCH4 has been shown to regulate nucleolar R-loops, controlling rRNA transcription through its interaction with DDX21 (Zhao et al., 2025). Regarding GPATCH8, this protein has been reported to fine-tune alternative splicing and to correct aberrant splicing events associated with mutant SF3B1. Furthermore, GPATCH8 and SUGP1 have been proposed to compete for DHX15 binding, thereby modulating DHX15 availability within the spliceosome (Benbarche et al., 2024; Cieśla and Bellodi, 2024). Finally, GPATCH11 has been identified as a multifunctional protein involved in RNA processing, splicing, and transcriptional regulation. Proteomic analysis has characterized its interactome, suggesting roles in RNA processing, splicing, and transcription regulation, and potentially also in synaptic plasticity and nuclear stress response (Zanetti et al., 2024).

Interestingly, several human G-patch proteins have been shown to perform multifunctional roles beyond RNA processing. For instance, AGGF1 has been reported to promote angiogenesis and vascular development by stimulating endothelial cell proliferation and migration, and to play protective roles in cardiovascular and muscle atrophy disorders (He et al., 2023; Lu et al., 2016). Intracellularly, AGGF1 has been found to act as a tumor suppressor by binding MDM2, stabilizing TP53, and regulating cell-cycle progression and apoptosis (Si et al., 2021). More recently, AGGF1 has been identified as a general splicing factor whose regulation of SRSF6 exon 3 skipping is essential for endothelial function and vascular development (Lv et al., 2025). On the other hand, G-patch protein NKRF has been described as a transcriptional repressor of NF-κB–responsive genes (Frattini et al., 1997). It has been shown to form a complex with DHX15 and the exonuclease XRN2, binding to transcribed spacer regions of pre-rRNA and stimulating early cleavage at site A′ during pre-rRNA maturation (Alexandrova et al., 2020; Memet et al., 2017).

On the other hand, human PINX1 has been shown to bind proteins TRF1 and TERT, thereby limiting telomere elongation, promoting senescence, and maintaining chromosome stability (Yuan et al., 2009). It has also been reported to interact with DHX15 via its G-patch domain to support ribosome biogenesis (Chen et al., 2014). Recent studies have revealed that PINX1 associates with UBTF and the RNA polymerase I subunit G, which is required for Pol I preinitiation complex formation (Lu et al., 2025). Furthermore, PINX1 has been demonstrated to interact constitutively with PARP1, enhancing chromatin association. This interaction promotes transcription of DNA repair genes such as *XRCC1* and transcriptional regulators such as *GLIS3*, thereby protecting cells from genotoxic stress (Huang et al., 2024).

Similarly, the G-patch protein GPKOW has been characterized as a key regulator of RNA metabolism. Its RNA-binding activity is modulated by phosphorylation through protein kinase A (Aksaas et al., 2011). Functionally, GPKOW has been demonstrated to be essential for efficient splicing and to suppress splicing defects caused by dominant-negative mutations in DHX16, underscoring its importance in maintaining splicing fidelity (Hegele et al., 2012; Zang et al., 2014). Regarding G-patch protein GPANK1, it exhibits low tissue specificity and cytoplasmic localization, with elevated expression in spermatocytes (Uhlén et al., 2015). Its precise molecular functions, particularly in RNA processing or helicase regulation, have yet to be defined. On the other hand, G-patch protein CHERP has been shown to regulate alternative splicing through interactions with U2 snRNPs and associated factors. Its depletion has been found to cause nuclear poly(A)^+^ RNA accumulation, intron retention, and cell-cycle abnormalities, underscoring its critical role in maintaining cellular homeostasis (De Maio et al., 2018; Martín et al., 2021; Wang et al., 2019; Yamanaka et al., 2022). Finally, G-patch protein CMTR1 has been shown to form a complex with DHX15 (Inesta-Vaquera et al., 2018). It functions as an mRNA cap methyltransferase, catalyzing 2′-O-ribose methylation of the first transcribed nucleotide of RNA polymerase II transcripts. CMTR1 is recruited co-transcriptionally to Ser5-phosphorylated Pol II CTD, where it binds transcription start sites and regulates gene expression and differentiation (Liang et al., 2022).

**FIGURE 2:**
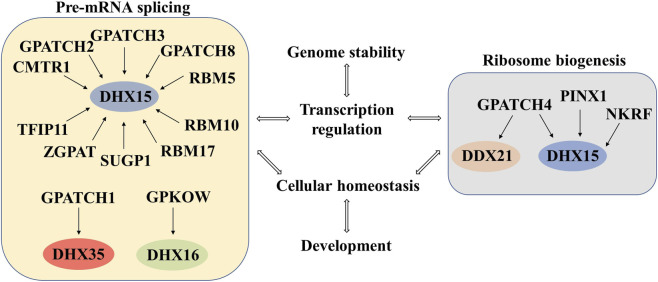
Roles of human G-patch proteins in RNA metabolism and cellular function. G-patch proteins interact with RNA helicases to coordinate pre-mRNA splicing and ribosome biogenesis. Dysregulation of these proteins can disrupt pre-mRNA splicing, ribosome biogenesis, transcription regulation, genome stability, and overall cellular homeostasis and development.

Summarizing, it is obvious that several human G-patch proteins function through interactions with their helicase partners, particularly DHX15, DHX16, DDX21, and DHX35, to regulate diverse RNA-processing pathways. Many, such as RBM5, RBM10, RBM17, GPATCH1, GPATCH2, GPATCH3, GPATCH8, SUGP1, and TFIP11, among others, directly modulate pre-mRNA splicing by controlling spliceosome assembly, alternative exon usage, or branch-point recognition, often stimulating helicase ATPase activity to ensure splicing fidelity and isoform diversity. Other G-patch proteins, including GPATCH4, NKRF, and PINX1, among others, interact with RNA helicases in ribosome biogenesis, rRNA modification, or transcriptional regulation, highlighting their multifunctional roles beyond splicing. Collectively, these observations emphasize a conserved mechanistic principle: G-patch proteins act as specificity factors that recruit and regulate RNA helicases, coupling enzymatic activity to distinct RNA-processing and cellular functions (Figure 2).

Building on these mechanistic insights, it is increasingly evident that dysregulation or mutation of human G-patch proteins contributes to disease. Proteins such as RBM5, RBM10, RBM17, GPATCH3, and GPATCH8 have been linked to aberrant splicing events that disrupt genes involved in apoptosis, cell-cycle control, and DNA repair, promoting tumorigenesis and cancer progression (Bechara et al., 2013; Cao et al., 2021; Fushimi et al., 2008; Ren et al., 2025). Similarly, alterations in TFIP11, GPATCH4, CMTR1, PINX1, and NKRF can impair ribosome assembly, rRNA modification, or transcriptional regulation, leading to defects in protein synthesis, genomic instability, and increased sensitivity to DNA-damaging stress (Chen et al., 2024; Zhao et al., 2025; Inesta-Vaquera et al., 2018; Yuan et al., 2009). Together, these few examples illustrate that disease-linked G-patch proteins converge on pathways essential for splicing fidelity, ribosome biogenesis, and genome stability, underscoring their relevance in cancer and other disorders and highlighting their potential as targets for therapeutic intervention.

Altogether, while the majority of human G-patch proteins primarily target spliceosomal helicases, recent studies reveal that their regulatory scope extends beyond pre-mRNA splicing. Increasing evidence suggests that these proteins act as versatile cofactors, coordinating helicase activity across distinct pathways and potentially linking RNA processing to chromatin organization, cellular stress responses and genome stability. To fully understand the molecular roles of human G-patch proteins and their complex networks of helicase interactions, further mechanistic and functional studies are needed that integrate biochemical, structural, and cellular approaches.

## 3 Conclusion and future perspectives

G-patch proteins are important cofactors of RNA helicases, orchestrating dynamic RNA-processing events including pre-mRNA splicing, ribosome biogenesis, transcription, and genome maintenance. Through these multifunctional roles, they integrate diverse RNA metabolic pathways, and their dysregulation can result in aberrant splicing, defective ribosome assembly, altered transcription, or genomic instability, with clear implications for human disease. Despite their evolutionary conservation, many G-patch proteins remain only partially characterized, and the principles governing their helicase specificity, regulatory mechanisms, and functional outcomes are still poorly understood. Key open questions include how G-patch proteins select specific RNA helicases in different cellular contexts, whether and how multiple G-patch proteins compete for the same RNA helicase, how individual proteins coordinate distinct RNA-processing pathways simultaneously, and which additional spliceosomal factors are regulated by G-patch proteins. Additional questions concern the molecular basis by which G-patch protein dysfunctions contribute to disease pathogenesis, particularly in cancer and disorders linked to splicing, ribosome biogenesis defects or genome instability. Addressing these questions will require integrated structural, biochemical, and cellular approaches to define G-patch interactomes, resolve mechanisms of RNA helicase recruitment and activation, and map their roles in regulating RNA metabolism. Ultimately, a deeper understanding of G-patch proteins promises to reveal fundamental principles of RNA metabolism and identify potential novel therapeutic targets.
